# Supporting Goal-Oriented Primary Health Care for Seniors with Complex Care Needs Using Mobile Technology: Evaluation and Implementation of the Health System Performance Research Network, Bridgepoint Electronic Patient Reported Outcome Tool

**DOI:** 10.2196/resprot.5756

**Published:** 2016-06-24

**Authors:** Carolyn Steele Gray, Walter P Wodchis, Ross Upshur, Cheryl Cott, Brian McKinstry, Stewart Mercer, Ted E Palen, Tim Ramsay, Kednapa Thavorn, Project Collaborators And Technology Partner, QoC Health Inc

**Affiliations:** ^1^ Bridgepoint Collaboratory Lunenfeld-Tanenbaum Research Institute Sinai Health System Toronto, ON Canada; ^2^ Institute of Health Policy, Management and Evaluation, Dalla Lana School of Public Health University of Toronto Toronto, ON Canada; ^3^ Toronto Rehabilitation Institute Toronto, ON Canada; ^4^ Institute for Clinical Evaluative Sciences Toronto, ON Canada; ^5^ Department of Family and Community Medicine Dalla Lana School of Public Health University of Toronto Toronto, ON Canada; ^6^ Department of Physical Therapy University of Toronto Toronto, ON Canada; ^7^ The Usher Institute of Population Health Sciences and Informatics Centre for Population Health Sciences University of Edinburgh Edinburgh United Kingdom; ^8^ School of Medicine University of Glasgow Glasgow United Kingdom; ^9^ Institute for Health Research Colorado Permanente Medical Group and Kaiser Permanente Institute for Health Denver, CO United States; ^10^ Ottawa Methods Centre Ottawa Hospital Research Institute The Ottawa Hospital Ottawa, ON Canada; ^11^ School of Epidemiology Public Health and Preventative Medicine University of Ottawa Ottawa, ON Canada

**Keywords:** eHealth/mHealth solutions, complex care needs, seniors, patient-centered care, goal-oriented care, primary health care, implementation, pragmatic trial, health outcomes, cost-effectiveness analysis

## Abstract

**Background:**

Older adults experiencing multiple chronic illnesses are at high risk of hospitalization and health decline if they are unable to manage the significant challenges posed by their health conditions. Goal-oriented care approaches can provide better care for these complex patients, but clinicians find the process of ascertaining goals “too complex and too-time consuming,” and goals are often not agreed upon between complex patients and their providers. The electronic patient reported outcomes (ePRO) mobile app and portal offers an innovative approach to creating and monitoring goal-oriented patient-care plans to improve patient self-management and shared decision-making between patients and health care providers. The ePRO tool also supports proactive patient monitoring by the patient, caregiver(s), and health care provider. It was developed with and for older adults with complex care needs as a means to improve their quality of life.

**Objective:**

Our proposed project will evaluate the use, effectiveness, and value for money of the ePRO tool in a 12-month multicenter, randomized controlled trial in Ontario; targeting individuals 65 or over with two or more chronic conditions that require frequent health care visits to manage their health conditions.

**Methods:**

Intervention groups using the ePRO tool will be compared with control groups on measures of quality of life, patient experience, and cost-effectiveness. We will also evaluate the implementation of the tool.

**Results:**

The proposed project presented in this paper will be funded through the Canadian Institute for Health Research (CIHR) eHealth Innovation Partnerships Program (eHIPP) program (CIHR–143559). The expected completion date of the study is November, 2019.

**Conclusions:**

We anticipate our program of work will support improved quality of life and patient self-management, improved patient-centered primary care delivery, and will encourage the adoption of goal-oriented care approaches across primary health care systems. We have partnered with family health teams and quality improvement organizations in Ontario to ensure that our research is practical and that findings are shared widely. We will work with our established international network to develop an implementation framework to support continued adaptation and adoption across Canada and internationally.

## Introduction

### Background: Understanding Seniors With Complex Care Needs and Their Challenges

Canadian and international health care systems require solutions on how to address the needs of a relatively small population that take up a large portion of health care resources. In Ontario, 10% of the population accounts for 79% of total system costs [1], with similar trends found in other parts of Canada [2] and internationally [3-5]. Most high-cost users are seniors, older adults, with multiple chronic conditions and complex care needs who are living in the community [6]. Beyond the cost issues, older adults experiencing multimorbidity are at higher risk of poor health outcomes and experience lower quality of life as compared with individuals experiencing a single illness only [7,8].

Understanding complex older adults, however, goes beyond how much they cost the health system or the number of chronic conditions they experience. It is important to understand the challenges faced by older adults from a bio-psycho-social perspective, which acknowledges broader social, environmental, and contextual issues that impact on the health care needs of these patients as well as their ability to manage [9]. A systematic review of the literature revealed that over half of the elderly population experiences multimorbidity, with a higher prevalence among those in the lower socioeconomic strata [7]. These social and contextual factors increase older adults’ vulnerability, which has been found to be associated with low quality of care delivery [10].

It is difficult for providers to support older adults with complex care needs because they have few (if any) tools, like clinical practice guidelines, to guide decision making [11]. This is problematic because providers are trying to help patients manage multiple conditions, many with discordant, competing symptoms and treatments that potentially run counter to each other [12]. It has been argued that these patients greatly benefit from patient-centered care approaches, which allow for individualized and holistic methods to address their highly variable needs [13,14]. At the provider level, patient-centered care approaches require a strong patient-provider relationship built on communication, respect, shared responsibility, and support for the patient as a whole person [13,15-17]. At the system level, patient-centered care can address poor care coordination issues experienced by complex patients [18].

Patient-centered care approaches are viewed as crucial to address the needs of this patient population [19,20], and can be supported by adopting goal-oriented care approaches [21]. Goal setting is also a key feature of coordinated care plans intended to support coordination and continuity of care for older adults, and others, with complex needs [22]. Goal-oriented approaches can help patients to prioritize their competing issues to help improve quality of life, while supporting primary health care providers and clinicians who have little evidence to draw on to support their older adult patients with complex care needs. However, clinicians find the process of ascertaining goals “too complex and too-time consuming” [23]. Additionally, goals are often not agreed upon between complex patients and their providers [24]. It is not surprising then that complex patients report not feeling engaged with their primary care provider in the management of their health conditions [25].

Primary health care providers require tools to overcome barriers to adopting goal-oriented care approaches to address the needs of a growing population of older adults with complex needs. Since April 2013, we have undergone a multiphased, user-centered design evaluation approach to develop the electronic patient reported outcomes (ePRO) mobile app and portal; a tool designed to meet the needs of older adults with complex care needs and their primary care providers. This study will evaluate the use, effectiveness, and monetary value of the ePRO tool through a cluster randomized controlled trial with an embedded case study of implementation and will answer the following primary research questions: (1) Does ePRO improve quality of life, care experience, and self-management in older adults with complex needs? (2) Is ePRO cost-effective for older adults with complex needs from the perspective of the health care system? (3) What are the most important implementation factors to effectively scale and spread ePRO in primary health care settings?

The ePRO tool’s focus on improving goal-oriented care for seniors experiencing complex chronic disease and disability living in the community marks a new contribution to the mHealth space. The proposed project will additionally provide instructive advances in rigorous evidence from a trial.

### The Electronic Patient Reported Outcomes Tool: An eHealth Solution for Community-Dwelling Complex Seniors

#### Electronic Patient Reported Outcomes Tool Development

To develop and test the ePRO tool, we have drawn on the principles of design research, which suggest that the development and adoption of technologies into real world environments requires an iterative approach where designs are progressively adjusted and refined based on emerging design principles, evolving needs, and end-user feedback [26,27]. User-centered technology development emphasizes the need to incorporate user feedback as part of the design, testing, and implementation process [28], while design research highlights the need to use rigorous research methods and evaluations in order to support capturing and incorporating user feedback [29]. We then iteratively engaged in end-user codevelopment of the tool through a mutliphased approach.

**Figure 1:**
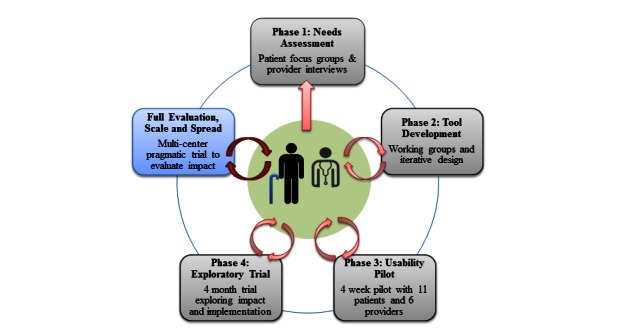
Development approach.

At the outset of the development process we broadly intended to develop a solution to support community-dwelling patients, including older adults, with complex needs and their providers in primary health care settings. Figure 1 provides a visual depiction of our iterative design and development method, which included patient, caregiver, and primary health care provider input at each phase. The proposed eHealth Innovation Partnerships Program (eHIPP) grant will support the final phase of this work, the full evaluation, scale, and spread phase depicted in Figure 1 (Adapted from [30]).

In Phase 1, we conducted a user-needs assessment using qualitative interpretive descriptive data collection and analysis methods. Focus groups were conducted with patients and caregivers with findings being published last year [31]. In Phase 2, we conducted a tool development phase in which we used findings from Phase 1 to develop a prototype, which was tested with three working groups: (1) patient and caregiver working group, (2) primary health care provider working group, and (3) expert and research team working group. The working groups assessed the feasibility and usability of the first prototype, and another round of redesign was conducted based on user and expert feedback [30]. We next conducted a usability pilot in Phase 3, with 11 patients and six primary health care providers from one Family Health Team in Toronto, Canada. The aim of the usability pilot was to determine the utility, functionality, and usability of the ePRO tool. Findings from this study informed further modification to the tool to improve usability [32]. The exploratory trial, Phase 4, began September 2015 and will be completed by August 2016.

#### The Electronic Patient Reported Outcomes Tool

The ePRO tool includes two features: My Goals and Outcome Measures. See Multimedia Appendix 1 for wire frames of the portal and mobile system.

##### Feature #1: My Goals

The My Goals feature allows patients and providers to collaboratively create goal-oriented patient care plans, and helps patients to track outcomes related to their goals using a mobile device. To set up goals patients (and caregivers if the patient chooses) sit down with their primary care providers during a visit in the physician’s office or in the patient’s home in the case of home visiting patients, and use a portal to create goals. Once a goal is added to the patients’ care plan, their mobile app will prompt them to report on outcomes related to that goal using the mobile device. Caregivers can enter data into the mobile device or on the portal on behalf of the patient if the patient requires assistance. Patients would either share their login information or caregivers can be given their own login to provide them with access. Specified-measureable- attainable-realistic-time specific (SMART) goal principles are used to guide goal set-up as this is an approach often used by primary care providers. We additionally incorporate goal-attainment scaling to create consistent goal attainment measures [33], which can then be used as a standardized patient outcome measure for patients with complex needs [21].

To create a SMART goal, the patient (with or without a caregiver) and primary care provider collaboratively specify the goal itself (eg, walk 20 minutes each day), the importance of that goal to the patient, perceived achievability of the goal, the timing of the goal (eg, achieved in 2 weeks), and any supports and resources available and/or required (eg, referrals needed or community services available like walking programs). Once the properties of the SMART goal are established, the patient and provider set up the monitoring protocol to allow the patient to report on their progress. Monitoring questions can be written by the patient and provider using question templates (ie, Likert scales, 10-point scales, visual analogue scales, open comment boxes, photos), or can be selected from the question bank created by the provider or any other provider in a single practice using the system.

In addition to the customizable monitoring questions, there are two established monitoring questions asked of each goal. The first asks patients to comment on how they are doing generally in relation to the goal to provide contextual information on their progress (patients or caregivers type into an open text box). The second is a modified standard goal attainment scale [33] to assess goal progress depicted in Table 1.

**Table 1:** Goal attainment scale monitoring.

Score	Goal achievement
+2	Much better than expected
+1	Better than expected
0	Goal (expected goal specified by patient and provider)
−1	Less than expected
−2	Much less than expected

Goal set-up can be completed in a 30-minute care planning appointment, which includes the time required to engage in a collaborative discussion between the provider and the patient to identify appropriate goals. Typically providers and patients will only focus on one or two goals at a time, which is more manageable for the patient.

##### Feature #2: Outcome Measures

The Outcome Measures feature is intended to help patients, their caregivers, and primary care providers to monitor patients’ symptoms and outcomes that were identified as important by patients with complex care needs in the first phase of our tool development [31]. Similar to the My Goals feature, once symptom monitoring is added to the patient’s Outcome Measures their mobile app will prompt them to report on symptoms on the mobile device.

The health status scales included were chosen in earlier stages of development of the tool based on: (1) patient, caregiver, and provider identification of the symptoms and outcomes most important for patients with complex needs to measure, (2) psychometric properties of the tools, and (3) relevance and demonstrated use of the tools in primary health care delivery. We included the patient reported outcome measurement information system (PROMIS) global health scale (GHS), pain interference, and health assessment questionnaires (HAQ) all of which are valid and reliable for patients with chronic illness [34-36]. The PROMIS and HAQ instruments have undergone rigorous validity and reliability testing, which include psychometric assessments as well as qualitative, quantitative, and mixed-methods research [36]. A systematic review of patient-reported outcome measurement tools funded by the Canadian Institutes of Health Research identified the PROMIS tools, in particular the GHS, to be among the more effective tools used to measure patient health outcomes [37]. The patient health questionnaire (PHQ9) and the generalized anxiety disorder (GAD7) tool were included as these are often used in primary health care practice, with demonstrated reliability and validity [38,39]. We also included monitoring protocols identified as important to these patients including: weight, blood pressure, heart rate, blood glucose, mood and emotion, sleep patterns, diet, and physical activity and walking logs. These protocols had been previously developed and tested with seniors by our technology partner.

Patients, their caregivers, and providers can track patient progress on goals and view their symptoms, vitals, and outcome data on the portal. Monitoring frequency is up to the patient and provider and can occur daily, weekly, or monthly depending on the goal or symptom being monitored. Patients are also able to track their progress on their mobile device and can then make adjustments to their routines if declines or low health status are indicated. Patients also have the option to monitor and track progress using the portal only rather than mobile device if they prefer to work on the computer. Providers can use monitoring data at the point-of-care to focus discussion on key patient concerns/issues, which can help to prioritize patient needs and support improved decision-making. As a clinician stated after participating in our usability pilot “you get that snapshot just before [the patient] comes in. You have a whole lot of data that is very efficient.”

#### Electronic Patient Reported Outcomes Tool Technology Readiness Level

The ePRO tool is at a Technology Readiness Level 7 as it has already undergone early implementation and acceptability testing through the usability pilot. By August 2016, we will have moved to a Level 8 after the completion of our exploratory trial in which we began integration of the system into family health teams (FHTs) with early testing of outcomes. The Could Connect platform used to deliver the ePRO solution is at a Technology Readiness Level 9, actual technology proven through operations. The Cloud Connect platform is fully built and has been validated in several studies with different patient populations. This readiness level has been validated through a vendor preapproval process through the Canadian Federal Government.

#### Electronic Patient Reported Outcomes Electronic Patient Reported Outcomes Data Security

All data captured within the ePRO tool and by extension on the QoC Health, Inc. platform, where the ePRO tool is housed, is secured through industry standard encryption mechanisms, which are compliant with Canadian and American data security standards as legislated under the Personal Information Protection and Electronic Documents Act, 2015 (in Canada) [40] and Health Insurance Portability and Accountability Act, 1996 (in the United States) [41].

#### Change Management and Implementation Plan

We will adopt Canada Health Infoway’s Change Management Framework [42] to support adoption of the ePRO tool for this study. This framework was designed to support the implementation of eHealth technologies, and identifies six core elements that should be addressed when implementing new eHealth solutions in health care settings including: governance and leadership, stakeholder engagement, communications, workflow analysis and integration, training and education, and monitoring and evaluation. These elements have guided our development to date as implementation considerations should be an ongoing process aligned with the development of eHealth solutions [43]. Table 2 provides an overview of the framework and how we have and will address each element as part of our development and implementation of the ePRO tool for this project. Our focus is on change management at the intervention sites with emphasis on organizational leaders and providers. In developing the ePRO tool, we have found that health care provider buy-in is among our greatest challenges and as such this will be a large focus of our change management strategy.

**Table 2:** ePRO tool change management strategy.

Change management element	Activities/strategies for adopting ePRO tool
Governance and leadership: mechanisms used to guide, steer or regulate the project. Also includes leadership activities in relation to change management.	With support from the decision-making partners and academic leads, site leads will be identified who will support the implementation process at the two intervention sites. These individuals will be engaged early in the process and will be informed about the value and need for the solution, including identifying the potential for the technology to improve outcomes, and reduce resource use. Decision-making partners and site leads will ensure that this message is shared with providers and other key stakeholders, will meet with the research team regularly, and will provide guidance on the change management activities outlined below.
Stakeholder engagement: activities that will support involvement by stakeholders expected to change. Behaviors must be defined, understood, and considered.	Stakeholder engagement with providers and patients has been integral to the design and development of the ePRO tool. We have learned that ongoing and continuous engagement with providers is particularly important to support uptake. As such we will: 1. Schedule several early meetings with providers at each site to introduce technology and field questions. 2. Leverage our civic partnerships as well as existing relationships with intervention sites to improve provider and patient engagement throughout the project.
Communications: how stakeholders will be informed of the change to initiate appropriate actions/behaviors.	We will use regular meetings, as well as our online messaging portal to track system errors, concerns, and suggestions for improvement from the research team to the technology partner (called the issue tracker), and short report/communications to update provider stakeholders on the progress of the project as needed. Providers, and patients, who are enrolled will have been provided with contact information for the team so they can easily share concerns, thoughts, and ideas on the tool at any time.
Workflow analysis and integration: understanding the current work process so that new tools can be sustainably embedded.	We will conduct a workflow analysis as part of this study to assess feasibility and usability of the ePRO tool into Ontario primary care practices. We will draw on methods and analysis from the workflow analysis, which was conducted as part of the usability pilot. Workflow analysis will be taken into consideration when interpreting our findings, and inform iterative changes to the tool to improve usability and uptake.
Training and education: activities needed to build capacity and skills among stakeholders expected to change.	Training and education are built into the research design. We plan on running at least one training session for providers and patients who are enrolled in the study and more as needed. Findings from the usability pilot indicated the need for ongoing information and potential training opportunities. As such we have developed user manuals for providers and patients. Refresher training sessions will be offered at 3, 6, and 9 months to all participants.
Monitoring and evaluation: reviewing and evaluating the change management process.	As part of our broader implementation plan we will include an evaluation of the change management process as part of our study. We will conduct readiness assessments prior to piloting, and will include questions regarding the change management approach in our follow-up interviews with organizational leaders and providers at intervention sites.

### Capacity for Integration of Electronic Patient Reported Outcomes Into Existing Models of Care

The ePRO tool is designed to work within existing models of primary health care in Ontario such as FHTs, which are made up of an interprofessional team of physicians, nurse practitioners, and other health care providers delivering primary health care services to patients [44]. The ePRO tool can further be adopted by any integrated primary health care teams and/or primary health care models adopting care coordination plans as part of usual care. The decision-making partners for this project, Health Quality Ontario (HQO) and the Association of Family Health Teams of Ontario (AFHTO), will support integrating ePRO into existing models of primary health care. HQO is mandated, in part, to support quality improvement of the primary care sector through supporting the adoption of a standardized care coordination plan among other change ideas. AFHTO is similarly dedicated to supporting the sharing and implementation of best practices to FHTs across Ontario as a means to deliver better health and value to patients.

## Methods

### Developmental Evaluation of the Electronic Patient Reported Outcomes Tool

To address our three research questions, we will conduct a pragmatic cluster randomized controlled trial with an embedded case study of implementation at four sites. We will adopt a developmental evaluation approach in which evaluation questions are used to support decision-making and modifications to improve interventions and programs [45]. As is consistent with developmental evaluations, we seek to capture outcome, process, and context measures to identify how the ePRO tool impacts on patient, provider, and system outcomes, identify processes and contexts that may be influencing outcomes, and implementation of the tool to support scale and spread. The literature on telemedicine assessment suggests the need to capture both the impact of interventions but also the contextual factors that influence outcomes [46]. Additionally, eHealth and mobile technology adoption can be understood as a complex health intervention [47], which are highly influenced by contextual factors [48]. Developmental evaluations such as ours proposed here are particularly important in assessment of complex systems [49].

### Setting and Site Recruitment

We will use FHTs in Ontario, Canada as cluster sites to test the ePRO tool. Working with AFHTO we will identify 22 FHTs that vary in terms of geographic location and then randomly assign sites as either intervention or control. Using our change management framework as a guide, we will seek ongoing engagement through weekly or biweekly communication with executive directors and lead physicians at each intervention and control site. Posters and pamphlets describing the technology and trial will be delivered to providers and patients at the intervention sites. Communication both written and electronic, including a question and answer portal system, will be used at both intervention and control sites to ensure ongoing engagement as a means to minimize loss to follow-up for comparative measurement.

### Sample Size Calculation

We will recruit 30 patients from each site, resulting in a total of 660 patients enrolled in the study. This sample size will provide 80% power derived from a power calculation based on a minimal clinically important difference of our core measure of quality of life (the assessment of quality of life, AQoL-4D) of 0.06 [50], an expected standard deviation in AQoL of 0.22 [51], an expected intraclass correlation coefficient of 0.01 (calculated based on total primary care use over a 1-year period among a 10% sample of the Ontario population, which served here as a proxy measure for patient outcomes), and an expected attrition rate of 10%. The expected attrition rate is derived from the recent study of the Change Foundation Partnership Advancing Transitions in Healthcare, which employed a very similar technology platform through a collaboration between QoC Health and the Health System Performance Research Network.

### Population and Patient Recruitment

Eligible patients will need to be rostered at FHTs recruited to participate, over age 65, have two or more chronic conditions, and have had 10 or more visits to their primary health care provider within the last 12 months. These variables have been associated with a high complexity score [52] and can be pulled from most primary practice electronic medical record (EMR) systems without the need for a full chart review. Given that the smallest FHTs have three or four physicians with a roster of at least 6000 patients, we anticipate more than 300 eligible patients in each practice from which to recruit patients, as has been found in the FHT practice where the ePRO tool was developed, therefore requiring no more than a 10% participation rate. Patients deemed eligible based on EMR records will be randomly selected and placed on an ordered list of eligible patients for recruitment into the study that will be used to recruit patients until our required complement of patients is enrolled at each team.

Patient recruitment will occur either during a scheduled visit or by phone within 1 month of the study start date. Administrators at the FHTs will seek permission from eligible patients to be contacted by a member of the research team. Contact information for patients who agree will be provided to the research team to obtain consent and enrolment. To enroll in the study, patients must: (1) be able to provide informed consent, (2) be willing to complete surveys at baseline, 3-, 6-, 9-, and 12-month intervals, (3) allow the researchers to extract health information from the EMR, (4) allow use of their health card number to link their study data to health administrative data, and (5) for patients at intervention sites, be willing to keep track of their health on an ongoing basis for a period of 12 months with an electronic device or via a Web-based portal if they are selected to be a part of the intervention group. Patients at intervention sites will also need to have the physical capability to use a tablet or smartphone or have a caregiver who is willing to use the device with them. At this time our tool is only English enabled so either the patient or caregiver will also need to speak and read English.

### Controlling for Bias and Contamination to Optimize Internal Validity

The clustered trial will control for contamination by clinical teams who are engaging with patients in setting goals with the ePRO in the intervention arm. We will ensure that providers who engage in this study do not provide care in multiple practices that are included in intervention and control arms of the study. Patient contamination should be prevented by selecting patients who are enrolled to the FHTs and have received most care within the team. Our random assignment of FHTs to intervention and control arms will occur after teams have consented to participate in the study; however, there may be a bias from teams who agree to participate from those who decline. We will compare participating from nonparticipating sites on practice geography, size (number of patients and number of physicians), and other characteristics based on membership information held by AFHTO. Patients who enroll in the study at intervention sites will likely be more technologically advanced than similar multimorbid patients who do not participate. We cannot measure technological literacy for nonparticipating patients at intervention sites but we will compare intervention and control patients to ensure comparability on study measures of technology use included in our baseline patient surveys. Patients in both intervention and control sites will also be asked to report on other disease management programs and activities that they are using. Similarly providers in both arms will be asked to report on other patient monitoring and goal-setting tools that they are using. We may expect that providers and patients already using such tools would have reasons to be both more but also less interested to participate in this study. The study design itself does not encourage bias in either direction.

### Training

Providers and patients recruited at intervention sites will receive training on how to use the ePRO tool prior to the start of the trial. Intervention patients will receive one-on-one training with a research assistant; this typically takes 30 minutes (as was found in our usability pilot). Provider training will occur in a group setting. Training will be led by a member of the research team and will take between 30 and 60 minutes. In our usability pilot, we found that a single training session was not sufficient for either patients or providers to fully learn to use the tool. As such, we will provide patients and providers with a manual and training video on how to use the tool and portal, and offer refresher training for patients at intervention sites at 3, 6, and 9 months.

### Outcome, Process and Context Measures

#### Pragmatic Trial Measures

The pragmatic trial will address research questions 1 and 2: does ePRO improve patient quality of life, care experience, and self-management in older adults with complex needs? Is ePRO cost-effective for older adults with complex needs from the perspective of the health care system? It should be noted that evaluation of the current tool will focus on whether the goal setting and patient use of the tool results in changes in health outcomes. We will not be able to assess whether changes in specific activity was associated with changes in health outcomes.

Patient quality of life, experience, and self-management will be captured using validated scales. Our primary measure of patient-oriented outcome is health-related quality of life measured by the AQoL-4D. The AQoL-4D takes only minutes to complete, has been validated in a community-dwelling older adult population, and has demonstrated responsiveness and predictive validity with regard to entrance to long-term care [51]. The AQoL-4D captures four core dimensions of health-related quality of life (independent living, relationships, mental health, and senses) that map closely onto factors that are identified in the ePRO tool [30]. Patient experience will be captured using measures from the patient-experience survey deployed to all FHTs from AFHTO and our other collaborating partner HQO. Using these measures would allow us to compare our findings with patient experience scores from FHTs across Ontario.

We will measure patient self-management using the 13-item patient activation measure (PAM) [53]. PAM classifies self-management capability into one of four categories ranging from only belief that their role as a patient is important, to certainty that they can take action even when under stress [54]. The PAM has been associated with better primary care experience [55] and improved health outcomes among multimorbid patients [56]. Finally we will look at goal-attainment scaling as captured by the ePRO tool for intervention patients, comparing outcomes captured at the start of the intervention to the end. Goal-attainment has been argued to be one of the most important outcome measures for complex patients [21].

With regard to our second research question, efficiency will be assessed using a cost-effectiveness analysis from health care system and societal perspectives. For the health care system perspective, only costs that are borne to the government, such as costs of intervention and costs of health services incurred during the 1-year follow-up period, will be considered. Costs of the intervention will be estimated based on anticipated real-world licensing and ongoing access costs for software, hosting and data management costs, program support, managed device costs, application support, training, incremental data plan costs (depending on volume of use in the intervention group), and costs for the Cloud Connect platform. These estimates will include any costs provided as in-kind contributions to the present study that would be recovered in a real-world adoption.

We will collect health care numbers from all participants and link all study measures with health administrative data (HAD) housed at the Institute of Clinical Evaluative Sciences (ICES; a research institute that collects a wide array of linked health datasets in Ontario). We will use HAD to follow total 1-year use and measure total direct costs to government for all health care services in both the intervention and control groups. Over 85% of total direct costs can be measured using a cost methodology for HAD implemented at ICES by WW [57]. For costs for which there is a service- or product-specific claim and a charge (eg, for prescriptions, fee for service physician visits) we will use the payment charge that is provided on those claims. For acute care hospitalization and emergency department (ED) costs we will multiply visit records in the Discharge Abstract Database and National Ambulatory Care Reporting System with encounter-specific resource intensity weights (also known as ambulatory cost weights in ED) and a provincial cost per weighted case.

Ontario also has separate databases to track post-acute rehabilitation and complex continuing care as well as inpatient mental health. In each case, we will use the appropriate resource intensity weight for that particular care setting but multiply, where necessary, by Ontario-specific weighted costs where these are not calculated and available through the Canadian Institute for Health Information. These cost weightings have been employed by our team in prior and current studies, following our costing methodology and using administrative data [1]. Capitation payments for primary care physicians in Ontario will also be calculated based on the payment rate and the particular model of primary care for each patient’s physician in each month of the study period. This method has been used in published studies of population-based health care costs [6,58].

From a societal perspective, administrative data will be combined with patient-reported health service use and costs (with patient-reported costs reported in the final 3-month period assumed to be representative for the last 6 months of the study) to provide an estimate of 1-year societal costs. Caregiver time costs will be estimated using the average industrial wage. We will also include patients’ and their caregiver(s’) time and expenditures related to health care for the past 3 months using measures that have been implemented as part of a standardized patient survey across 12 primary care research teams in the Canadian Institute for Health Research (CIHR) Community-Based Primary Health Care Team Grant competition (WW is a primary investigator for one team using this tool).

We will capture patient and provider demographic and characteristic information such as age, gender, ethnicity, chronic illness profile, socioeconomic status, and information technology (IT) skills to provide contextual information about our users to support our analysis. From the patient perspective, this will allow us to do case matching to control group. These contextual factors have also been found to impact the adoption and implementation of eHealth tools [59] and as such should be included in our analysis of barriers and facilitators.

#### Case Study Measures

Our third research question (What are the most important implementation factors to effectively scale and spread ePRO in primary health care settings?) will be answered through our case study design. A subset of four intervention sites will be selected to capture process and additional context measures to develop an implementation framework to support scale and spread of the ePRO tool. Cases will be selected based on a most different design, ensuring that we capture practices that differ in terms of location (rural vs urban) and organizational design (ie, the number of diversity of providers at the practice). Implementation relates to the processes required to put an intervention or new model of care into use [60]. Implementation factors are important to assess to determine how best to scale and spread (ie, increasing coverage, range, and sustainability [61] of the ePRO tool). In terms of scale, we are interested in what additional features/capabilities could be added to the tool to meet patient and provider needs not yet addressed, and in terms of spread we seek to determine provider, organizational, and health system enablers and barriers to adopting the tool in primary care practices broadly across Canada and internationally.

Given the importance of patient-centered care delivery for seniors with complex care needs we will additionally capture provider level effectiveness through provider interviews guided in-part by the Assessment of Chronic Illness Care (ACIC) tool. The ACIC has been used to help health care teams improve care delivered to patients with chronic illness [62], and it can also be used to measure change in care delivery. We will draw on the self-management and clinical information systems scales of the ACIC to craft interview questions as these are most relevant to our intervention.

Process measures will be captured through the post-study system usability questionnaire (PSSUQ) to assess use and experience using the ePRO by patients and providers. The PSSUQ is a 19-item usability questionnaire comprised of three subscales (system usefulness, information quality, and interface quality) [63]. The PSSUQ has demonstrated reliability and validity [64], and has been used to assess satisfaction and experience with similar mHealth technologies [63], which are key aspects of innovation model testing [65]. Patient and provider experience with the ePRO will additionally be captured through patient focus groups and provider interviews.

We will capture organization and system level context measures related to implementing the ePRO through post-intervention interviews with providers and organizational leaders. Factors such as supportive resources (ie, IT support), logistical issues (ie, integration of the tool into provider workflows), appropriate training and time to learn new systems, and organizational level support have been found to be pivotal in adopting new eHealth systems [59,66,67]. These factors are reflected in the change management framework used to guide development and implementation of ePRO (outlined in Table 2). The framework will be used to help shape interview guides and inform analysis. System level factors found to impact eHealth adoption, such as noncentralized systems, lack of standardization of data systems, legal requirements, and financial incentives (or disincentives) [66], will also be captured.

Outcome, process, and context measures that will be captured as part of the pragmatic trial and case study are outlined in Table 3 below.

**Table 3:** Outcome, process, and context measures for the developmental evaluation of ePRO.

Concept	Measurement level	Variable	Tool/method	Data collection
**Outcome: intervention and control sites**				
	Patient	Quality of Life	AQoL-4D^a^	Baseline, 3, 6, 9, and 12 months
		Self-management	PAM^b^	Baseline, 3, 6, 9, and 12 months
		Patient experience	Patient experience survey (from AFHTO^c^ and HQO^d^)	Baseline, 3, 6, 9, and 12 months
		Goal-attainment captured by ePRO tool–intervention sites only	Goal attainment scaling. Completed as part of the intervention.	Over 12 months
	System	Efficiency	Cost-effectiveness analysis: data from AQoL-4D, ICES^e^, patient self-report, and published literature	Pre and post-intervention
**Process: intervention sites**				
	Patient	Tool experience	PSSUQ^f^ post-study system usability questionnaire	3, 6, 9, and 12 months
			Patient focus groups	6 months and post-intervention
	Provider	Tool experience	PSSUQ^f^	6 and 12 months
			Provider interviews	6 months and post-intervention
		Delivering patient-centered care	Provider interviews–drawing on Assessment of Chronic Illness Care tool	6 months and post-intervention
	Organization	Provider workflows	Provider interviews	Post-intervention
**Context: intervention and control sites**				
	Patient	Demographic characteristics	EMR^g^ extraction; patient information sheet	Baseline
	Provider	Demographic characteristics	Provider information sheet	Baseline
	Organization	Size; description of the organization; resources; support; training	Document analysis; provider and leaders interviews	6 months and post-intervention
	System	Structure; standardization of data systems; legal requirements; funding	Document analysis; provider and leaders interviews	6 months and post-intervention

^a^Assessment of Quality of Life-4D

^b^patient activation measure

^c^Association of Family Health Teams of Ontario

^d^Health quality Ontario

^e^Institute of Clinical Evaluative Sciences

^f^Post-study system usability questionnaire

^g^electronic medical record

### Data Collection

**Figure 2:**
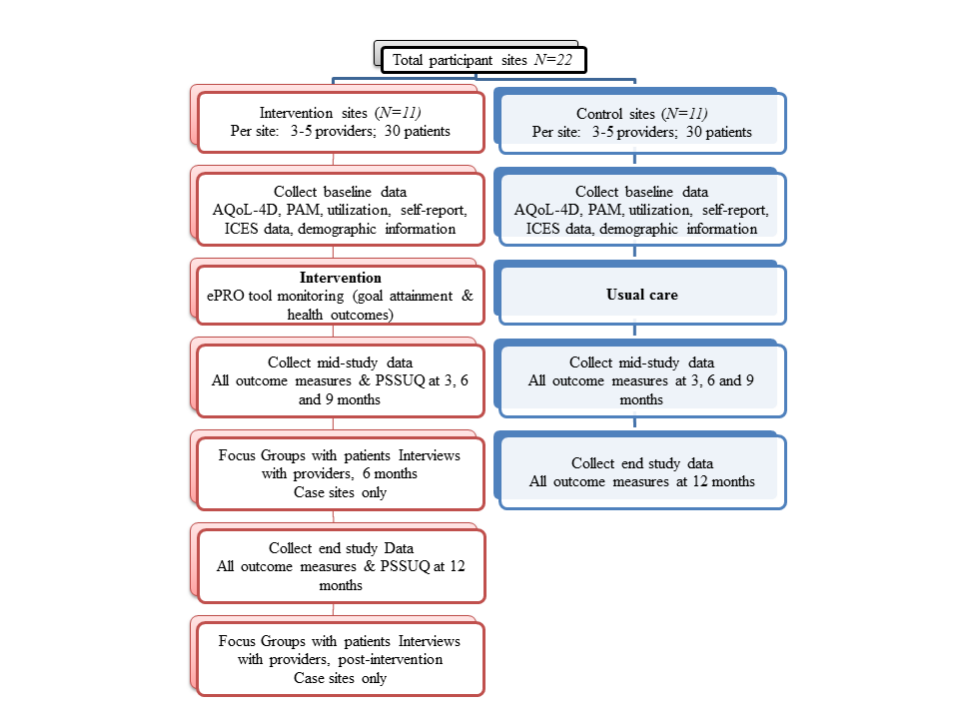
ePRO evaluation data capture diagram.

As can be noted from the Table 3 data collection will happen at baseline, 3-, 6-, 9 and 12-month time-points in the study, as well as post intervention (within 2 months of the end of the trial) at case study sites. Patients will not be asked to complete all surveys in a single sitting and will be given up to 2 weeks to complete surveys with the help of a research assistant at a location of the patients choosing (ie, in their home or at their FHT) at each data collection time point. Qualitative data collection will occur at 6 months and post intervention as four case study sites. Patient focus groups will have between three and four focus groups at each site with six to eight patients and caregivers participating in each. Keeping focus groups to a maximum of eight participants is a standard recommendation in focus group methodology [68] as it provides ample opportunity for each participant to voice their insights and perspectives. Focus groups will last between 60 and 90 minutes. Interviews will be conducting with the providers participating at the four case study sites and will last up to 60 minutes. Figure 2 visually depicts the data collection method.

### Data Analysis Strategy

Our data analysis strategy is broken up into our pragmatic trial analysis and case study analysis. The pragmatic trial analysis focuses on answering research questions 1 and 2 while our case study analysis will address question 3.

#### Pragmatic Trial Analysis

Comparisons between intervention sites and control sites across measures of location, practice size, academic affiliation, and other practice measures will be conducted. Similarly, we will compare patients on all baseline measures of age, sex, morbidity, socioeconomic circumstances, social roles (ie, caregiver availability and responsibility) and ethnicity, IT capability, and experience. Cohen’s-D and statistical differences will be used to determine differences in practice and patient measures that may need to be controlled for in statistical regressions if there are any such differences.

Statistical analyses will be undertaken to address the research study question 1. ePRO tool effectiveness will be determined by analyzing patient outcome data. Overall domain scores for AQoL-4D, patient experience, and PAM, will be calculated, including changes in scores between baseline and follow-up periods within groups and between intervention and control groups. Statistical comparisons between the intervention and control groups will be made using mixed-effects regression models to account for the clustering effect of patients within FHTs.

To address research study question 2, total cost for each patient including costs of the intervention and costs of health services over the 1-year follow-up period after the start of the intervention will be compared between intervention and control groups. Additional analyses will use patient-reported costs to estimate costs from the societal perspective. Results of the cost-effectiveness analysis will be expressed as the incremental cost per quality-adjusted life years (QALYs) gained, calculated as the difference in costs divided by the difference in QALYs between intervention and control groups. QALYs will be calculated using the total area under the curve approach, with linear interpolation between assessment points and baseline adjustment for comparisons [69]. We will use mixed-effects regression analyses to separately estimate the difference in health care costs between the intervention and control groups, and include any covariates that are observed to be different between groups in baseline comparisons.

In addition, we will calculate an incremental net benefit (INB) by subtracting incremental costs from a product of willingness to pay and incremental health benefits. If the INB is greater than zero, ePRO is considered as a cost-effective option. The 95% confidence interval will be calculated using a nonparametric bootstrapping method. Results from the simulations will also presented as cost effectiveness acceptability curves, which show the probabilities that ePRO being cost-effective over a range of potential willingness to pay [70]. We will conduct a scenario analysis, whereby the effect of a complete case only will be used to estimate the cost-effectiveness. Because of the 12-month follow-up during the study, costs and health outcomes will not be discounted. We will also conduct a budget impact analysis to estimate the financial consequences of implementing ePRO for a primary care provider and at the system level across a province depending on the number of primary providers.

#### Case Study Analysis

Single case and cross-case comparative analysis will be used to assess process and outcome measures captured at intervention sites in order to answer research question 3. Experience with the tool and feasibility of adopting the tool will be captured by analyzing data from the PSSUQ, patient focus groups, and provider interviews. Standard descriptive statistics will be used to analyze the PSSUQ across the three subdomains captured by the tool and comparisons between intervention sites will be made using *t* tests or Mann-Whitney tests as appropriate.

Focus group, interview, and field note data will be analyzed using qualitative descriptive methods [71]. Focus groups and interviews will be recorded and transcribed verbatim by an external source. Transcripts will be checked for accuracy against the audio record and analyzed through the assistance of NVivo 11 software. Two researchers trained in qualitative research will read the transcripts and record key themes, compare, and discuss findings.

Through comparing findings across the four FHTs and by engaging international research partners to discuss implementation factors of health care systems in Scotland and at Kaiser Permanente Colorado, we will aim to refine the change management and implementation frameworks used to guide the study to inform the development of an implementation strategy for the ePRO tool internationally.

## Results

The proposed project presented in this paper will be funded through the CIHR eHIPP program (CIHR–143559). The expected completion date of the study is November, 2019.

## Discussion

### Impact

#### Anticipated Outcomes From Adopting the Electronic Patient Reported Outcomes Tool

Using innovative technology to support the adoption of goal-oriented care for older adults with complex needs in primary care settings can have a significant impact on patient, provider, and health system outcomes. Our primary outcome of interest at the patient level is quality of life. For older adults with complex needs and their caregivers, an easy to use tool that supports goal-oriented patient care can support individualized discussions between providers and patients, can simplify patient- and joint-decision making processes, particularly for individuals with multiple chronic conditions, and can help patients to understand and articulate their needs [21]. By supporting goal-oriented care, the ePRO tool can enhance the quality of primary health care delivery by improving patient-provider interactions at the point of care, which has been found to improve health-related quality of life, and support positive health behavior change in chronic disease patients [72].

For health care providers the tool can help to address the challenges associated with delivering goal-oriented care [23,24], while providing patient-centered data to help make decisions about their care. The system can help providers to improve chronic illness management for their older adult patients with complex care needs by giving them a tool to help support patient self-management, as well as offer a clinical information system that provides monitoring data to help manage these patients; two key aspects of chronic illness care management in Wagner’s Chronic Care Model [73].

For the health system, our tool has the potential to increase access to primary health care services through mobile monitoring, and reduce unnecessary health care use. Our usability findings show early evidence of the tools ability to support patient self-management, which has been shown to help avoid declines and unnecessary health care use for patients with chronic disease [74,75]. With scale and spread of the tool it could further support system integration by allowing providers across the system to communicate about patient care plans, goals, and outcomes, which could lead to better management and slower decline over the longer term.

### Anticipated Ethical, Social, and Legal Issues That May Arise

Given the multiphased nature of our design, we have received four separate ethics approvals from all appropriate Research Ethics Boards to develop and test our tool. Among concerns we have addressed are (1) data security, which is addressed by ensuring our tool is PHIPA compliant, (2) provider liability for monitoring, which is addressed by ensuring participants are aware that the tool is not an emergency device nor is it monitored on a regular basis by providers, and (3) ensuring providers do not deny needed care. This last issue is addressed as our studies do not require that control patients be denied engaging in goal setting or monitoring with their providers should it be done as part of their usual care. We can draw on our experience running the usability pilot and exploratory trial to ensure we address, monitor, and evaluate our performance in relation to these important issues.

### National and International Scalability of the Electronic Patient Reported Outcomes Tool

We have developed and tested the ePRO tool in FHTs, an interprofessional primary care delivery model prominent in Ontario [44]. There are currently 184 FHTs across Ontario serving over 3 million Ontarians in over 200 communities [76]. The ePRO tool could be rolled out to any of these FHTs, and would be particularly useful to those serving older adults with complex care needs. Furthermore, as we designed the ePRO tool to align with the goal-setting section embedded in the Coordinated Care Plan, the tool can be scaled further to any primary care team adopting Coordinated Care Plans as part of their management of chronically ill patients. Additionally, the Coordinated Care Plan being used in Ontario is derived from common elements found in care planning from other jurisdictions including: British Columbia, Nova Scotia, Ireland, Scotland, England, the Netherlands, Sweden Australia, and the United States [22], suggesting the potential for international applicability.

International scalability is further supported through our partnerships developed with support from a CIHR Planning and Dissemination Grant awarded in 2014. The grant supported the development of a partnership between the Health System Performance Research Network and the Bridgepoint Campus of the Lunenfeld-Tanenbaum Research Institute (formerly Bridgepoint Collaboratory for Research and Innovation) in Ontario, with Kaiser Permanente Colorado in the United States and the Universities of Glasgow and Edinburgh and the National Health Service in Scotland. The international partnership was founded on a shared interest in supporting older adults with complex care needs in primary care settings in all three countries. Through site visits, and knowledge sharing via team teleconferences and reporting we identified an interest and opportunity to adopt the ePRO tool in Scotland and Kaiser Colorado, and plan to run feasibility pilots in these two settings. Findings from the pilots will further support spread and scalability of the tool nationally and internationally.

## Appendix

Multimedia Appendix 1 The ePRO tool.

Multimedia Appendix 2 eHIPP reviewer comments.

## Abbreviations

ACIC: assessment of chronic illness tool
AFHTO: Association of Family Health Teams of Ontario
AQoL-4D: assessment of quality of life measure
CIHR: Canadian Institute for Health Research
ED: emergency department
eHIPP: eHealth Innovation Partnerships Program
EMR: electronic medical record
ePRO: electronic patient reported outcomes
FHT: family health team
GAD7: generalized anxiety disorder
GHS: global health scale
HAD: health administrative data
HAQ: health assessment questionnaires
HQO: Health Quality Ontario
ICES: Institute of Clinical Evaluative Sciences
INB: incremental net benefit
IT: information technology
PAM: patient activation measure
PHQ9: patient health questionnaire
PROMIS: patient reported outcome measurement information system
PSSUQ: post-study system usability questionnaire
QALYs: quality-adjusted life years
SMART: specified-measureable-attainable-realistic-time specific
