# Correction of: Supporting Goal-Oriented Primary Health Care for Seniors with Complex Care Needs Using Mobile Technology: Evaluation and Implementation of the Health System Performance Research Network, Bridgepoint Electronic Patient Reported Outcome Tool

**DOI:** 10.2196/resprot.6719

**Published:** 2016-10-13

**Authors:** Carolyn Steele Gray, Walter P Wodchis, Ross Upshur, Cheryl Cott, Brian McKinstry, Stewart Mercer, Ted E Palen, Tim Ramsay, Kednapa Thavorn, Project Collaborators And Technology Partner, QoC Health Inc

**Affiliations:** ^1^ Bridgepoint Collaboratory Lunenfeld-Tanenbaum Research Institute Sinai Health System Toronto, ON Canada; ^2^ Institute of Health Policy, Management and Evaluation, Dalla Lana School of Public Health University of Toronto Toronto, ON Canada; ^3^ Toronto Rehabilitation Institute Toronto, ON Canada; ^4^ Institute for Clinical Evaluative Sciences Toronto, ON Canada; ^5^ Department of Family and Community Medicine Dalla Lana School of Public Health University of Toronto Toronto, ON Canada; ^6^ Department of Physical Therapy University of Toronto Toronto, ON Canada; ^7^ The Usher Institute of Population Health Sciences and Informatics Centre for Population Health Sciences University of Edinburgh Edinburgh United Kingdom; ^8^ School of Medicine University of Glasgow Glasgow United Kingdom; ^9^ Institute for Health Research Colorado Permanente Medical Group and Kaiser Permanente Institute for Health Denver, CO United States; ^10^ Ottawa Methods Centre Ottawa Hospital Research Institute The Ottawa Hospital Ottawa, ON Canada; ^11^ School of Epidemiology Public Health and Preventative Medicine University of Ottawa Ottawa, ON Canada

The authors of “Supporting Goal-Oriented Primary Health Care for Seniors with Complex Care Needs Using Mobile Technology: Evaluation and Implementation of the Health System Performance Research Network, Bridgepoint Electronic Patient Reported Outcome Tool” (JMIR Res Protoc 2016;5(2):e126) would like to make changes in the results section of the abstract as well as in the results section of the body of the article. In both instances the CIHR funding number should read: (CIHR-143559) instead of (CIHR-348362).

The authors would also like to change the funding number in the acknowledgements section with regard to the CIHR Planning and Dissemination Grant. The bracket should read (CIHR-137200) instead of (CIHR-328229). 

The authors would also like to change the order of affiliations 1 and 2 so that “Institute of Health Policy, Management and Evaluation, Dalla Lana School of Public Health, University of Toronto, Toronto, ON, Canada” becomes affiliation 2 and “Bridgepoint Collaboratory, Lunenfeld-Tanenbaum Research Institute, Sinai Health System, Toronto, ON, Canada” becomes affiliation 1. This changes the order of affilition for author Walter Wodchis from 1,3,4 to 2,3,4. The affiliation of author Ross Upshur is now rearranged from 2,5 to 1,5 and the affiliation of Project Collaborators And Technology Partner, QoC Health Inc is now 1 which was previously 2.

The last and final correction is that the authors would like to change their email address from csteele@bridgepointhealth.ca to carolyn.steelegray@sinaihealthsystem.ca.

These changes have been corrected in the online version of the paper on the JMIR website on October 13, 2016 together with publishing this correction notice. A correction notice has been sent to PubMed, and the publication was resubmitted to PubMed Central and other full-text repositories.

