# Supplementary figures and images for: Supporting Goal-Oriented Primary Health Care for Seniors with Complex Care Needs Using Mobile Technology: Evaluation and Implementation of the Health System Performance Research Network, Bridgepoint Electronic Patient Reported Outcome Tool
Source: JMIR Res Protoc. 2016 Jun 24;5(2):e126. doi: 10.2196/resprot.5756 (PMC4938886; doi:10.2196/resprot.5756)

## Slide 1
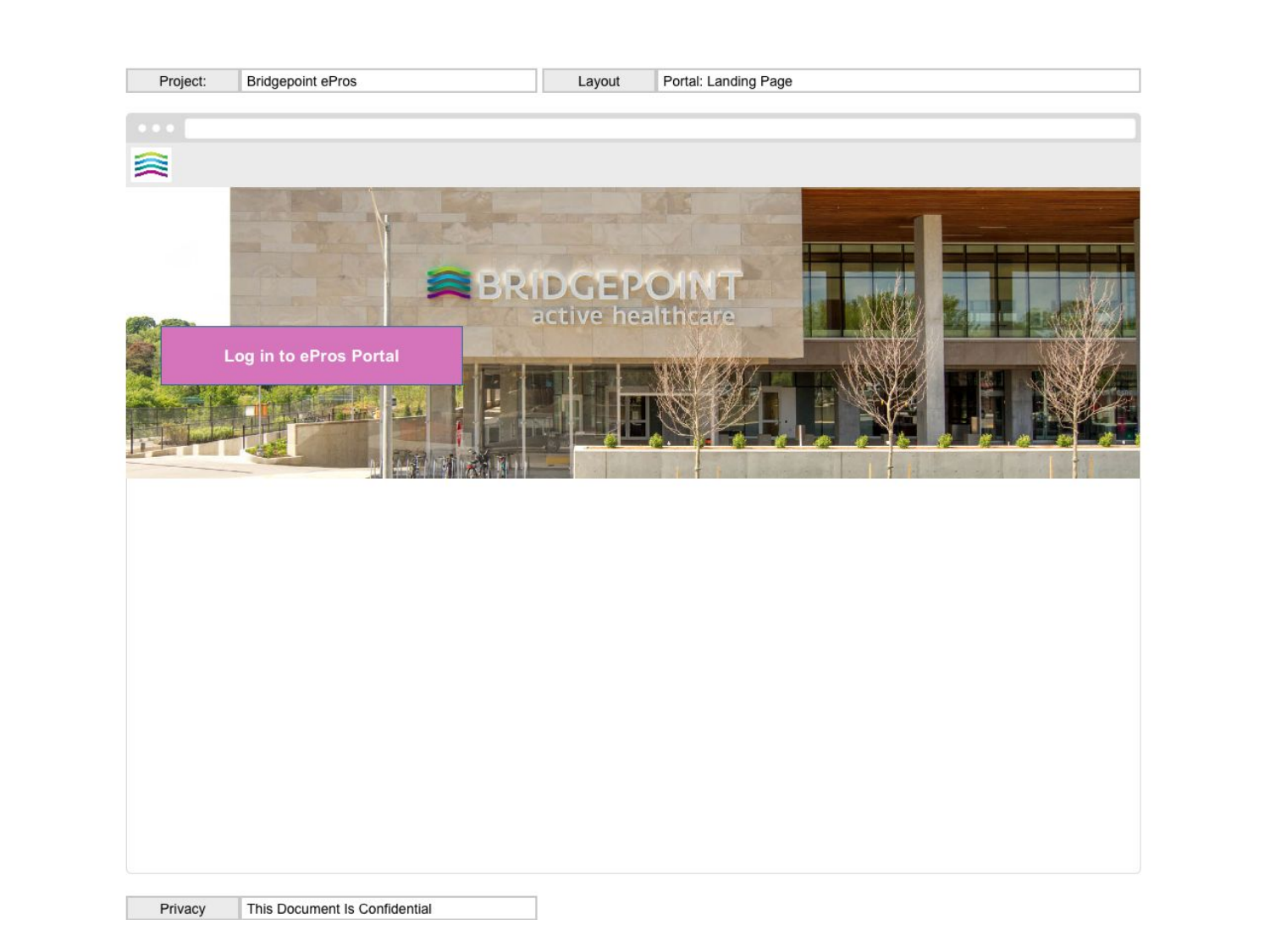

## Slide 2
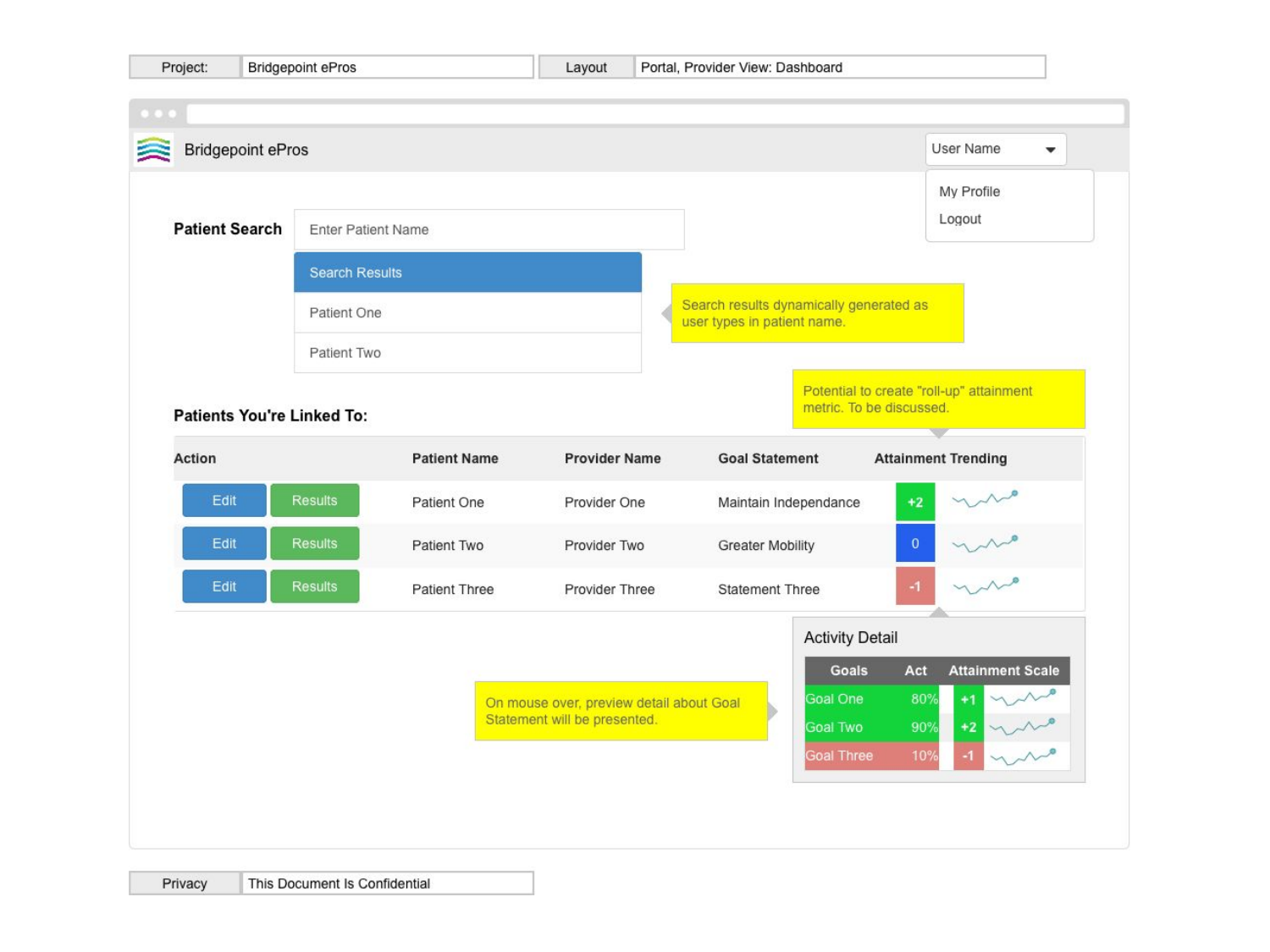

## Slide 3
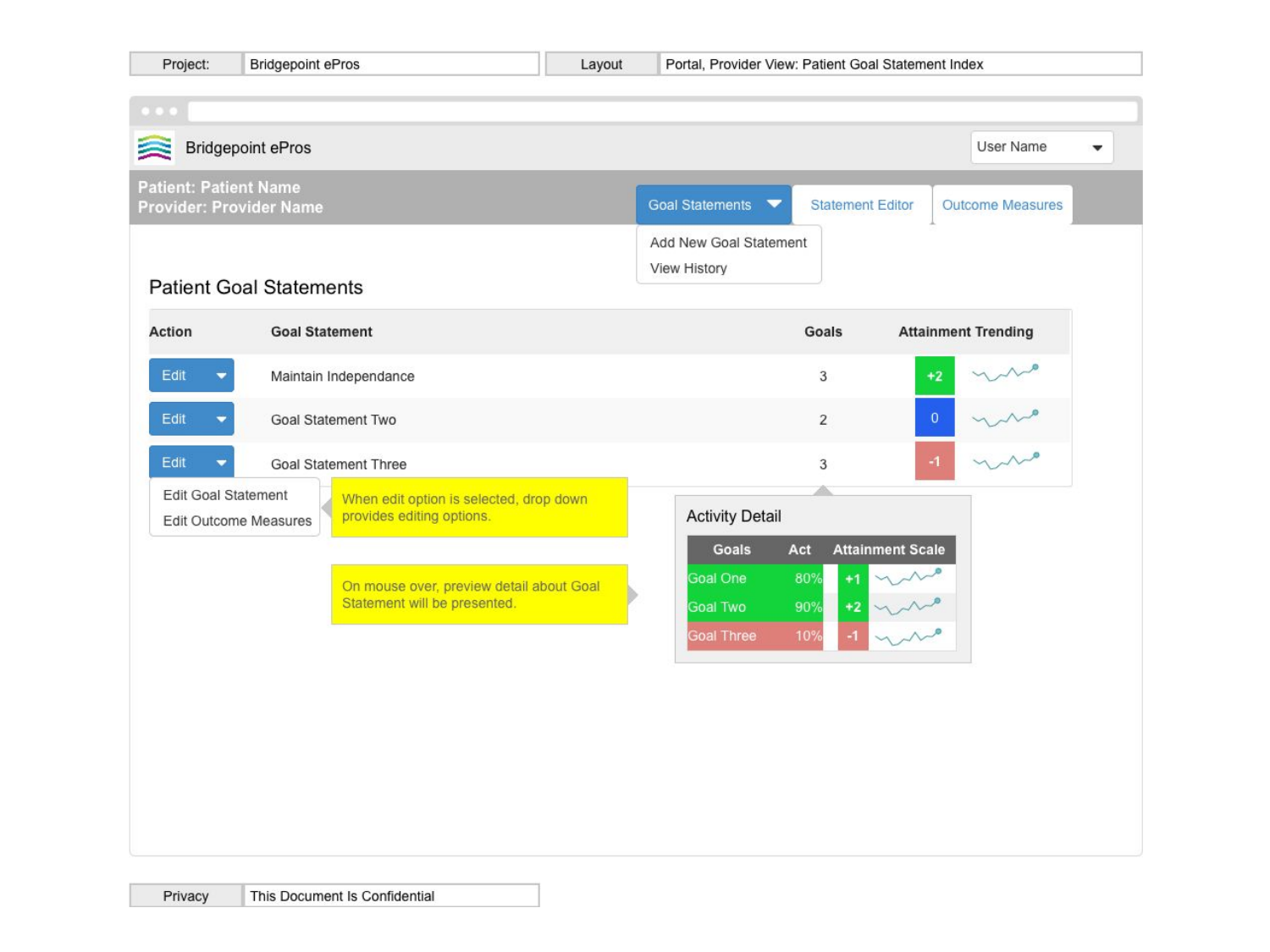

## Slide 4
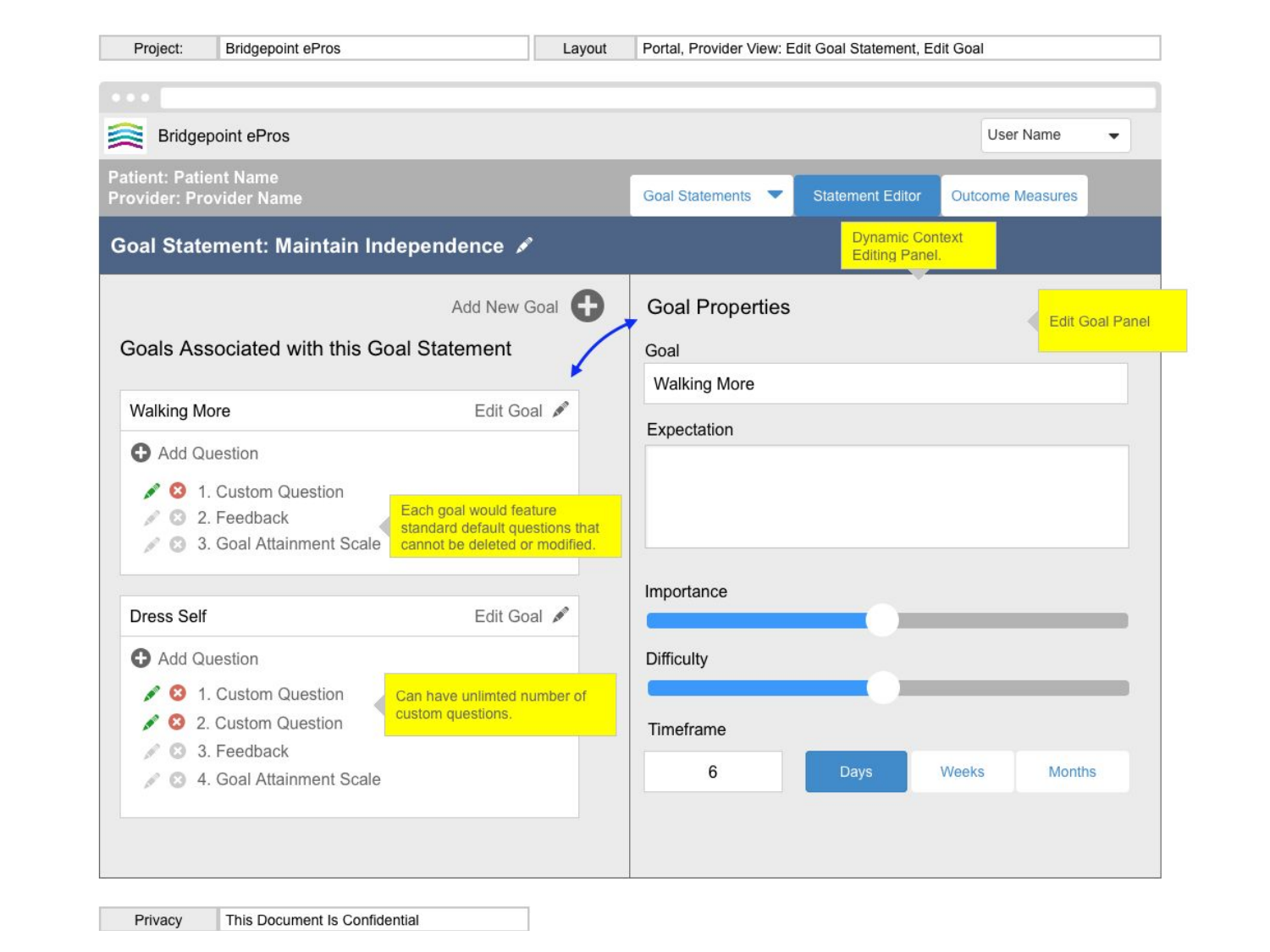

## Slide 5
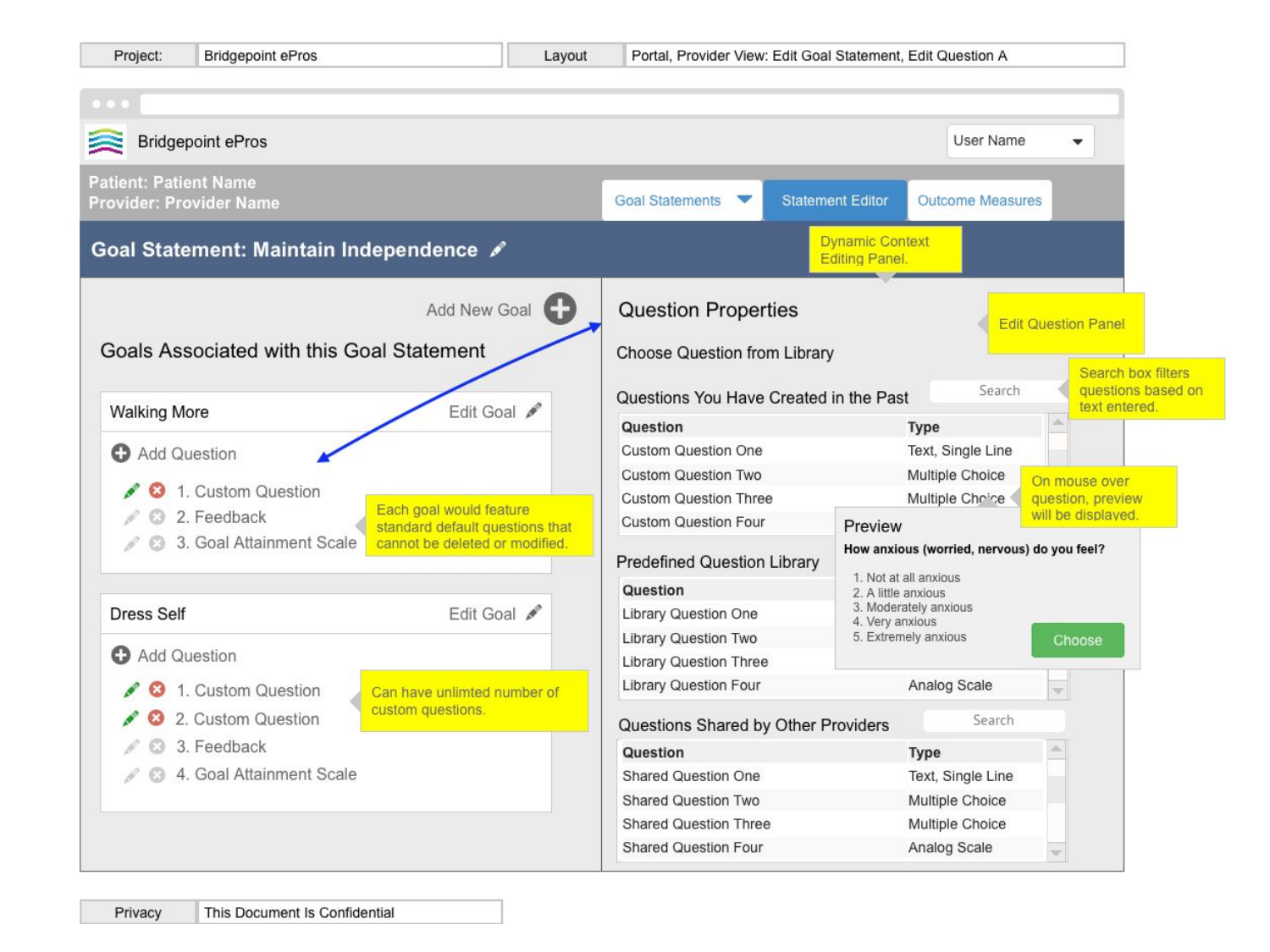

## Slide 6
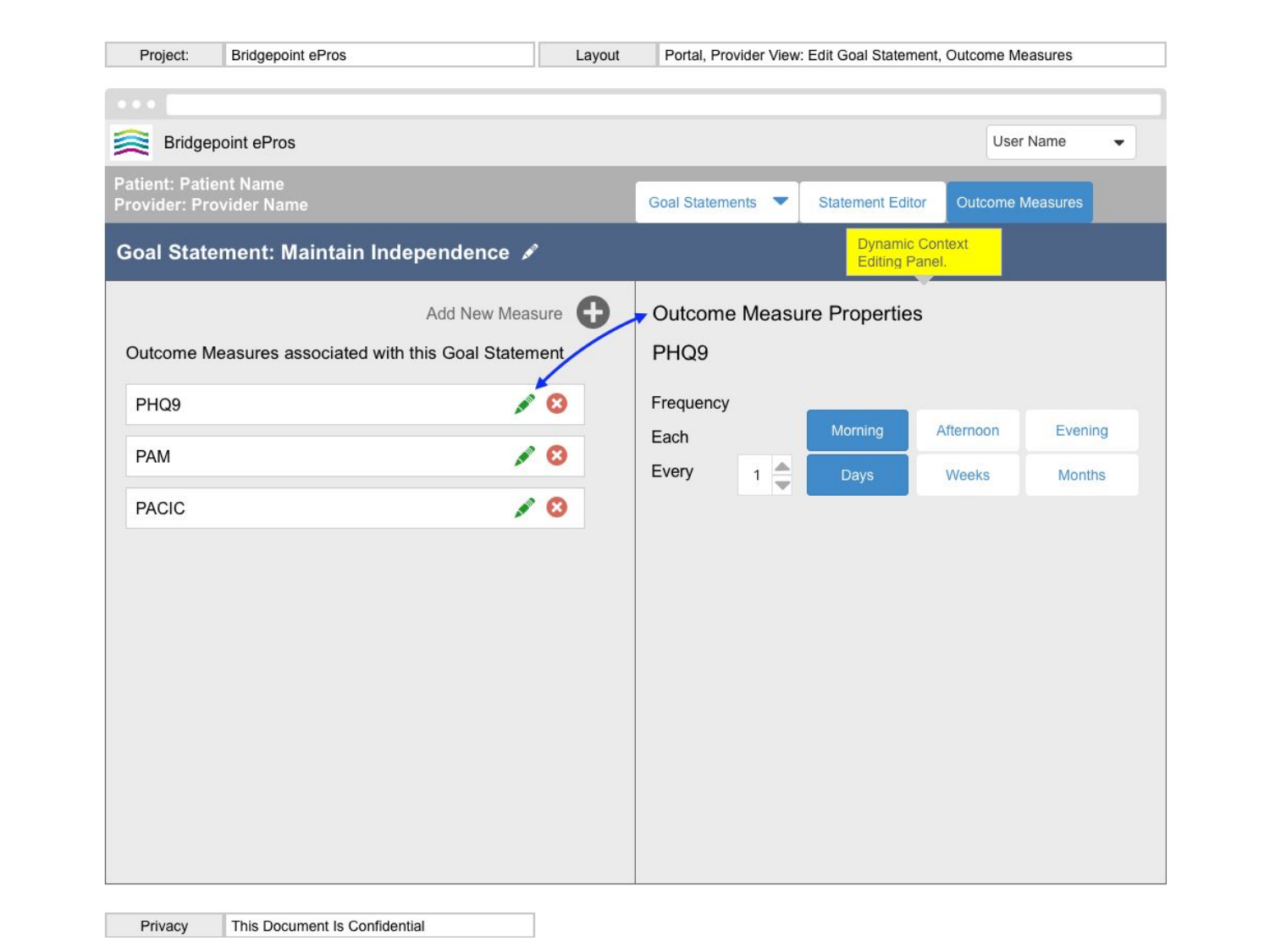

## Slide 7
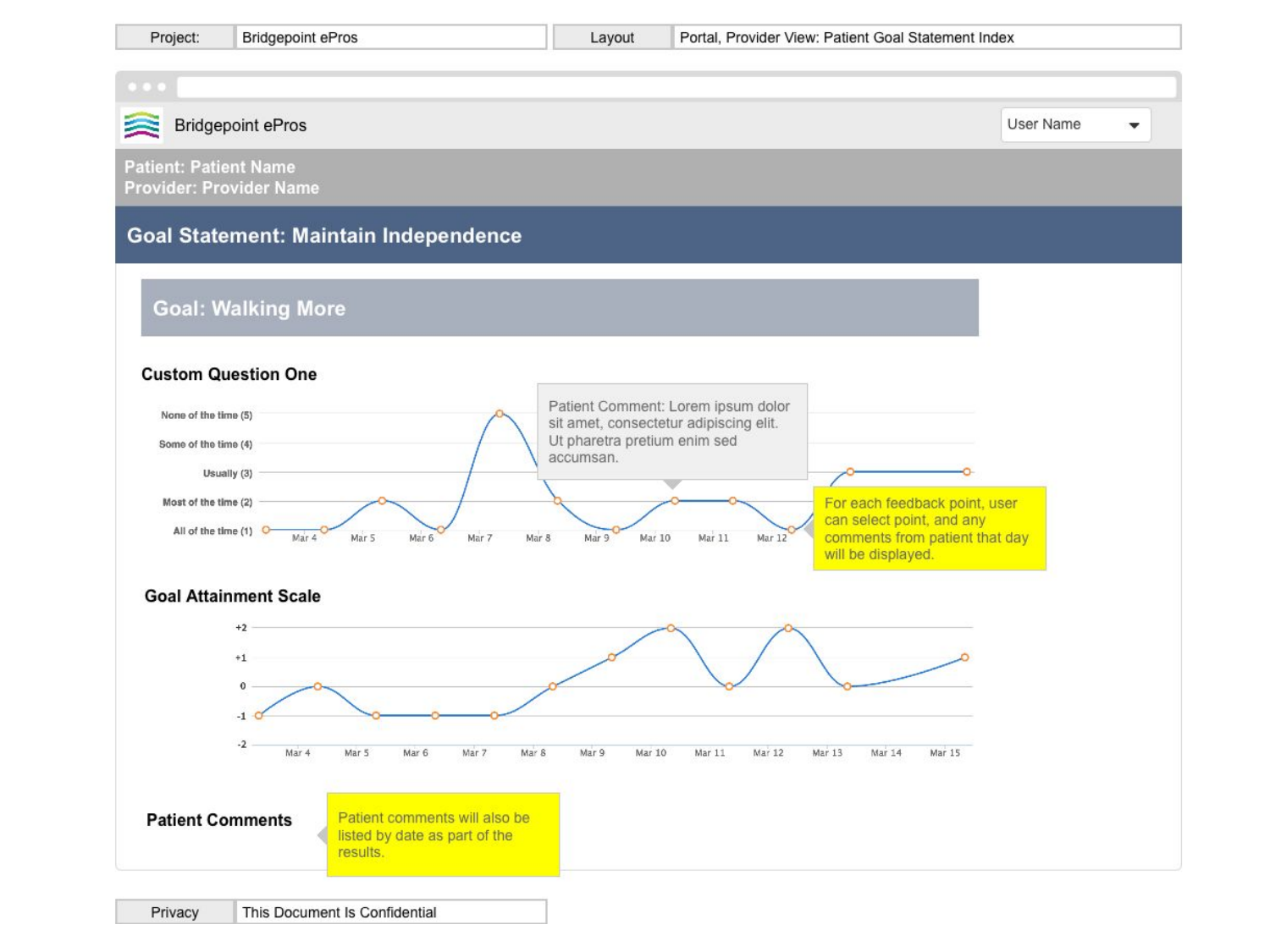

## Slide 8
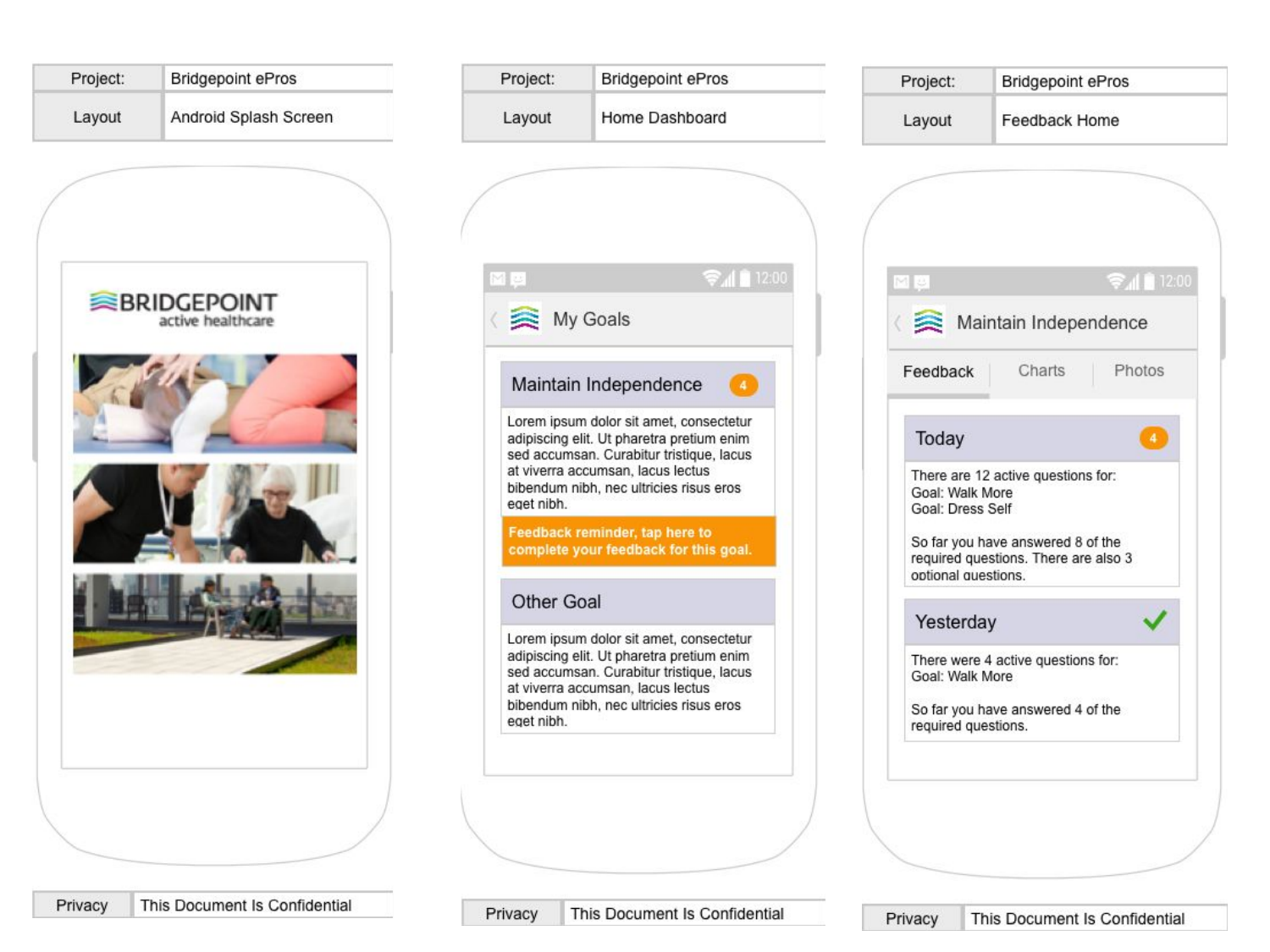

## Slide 9
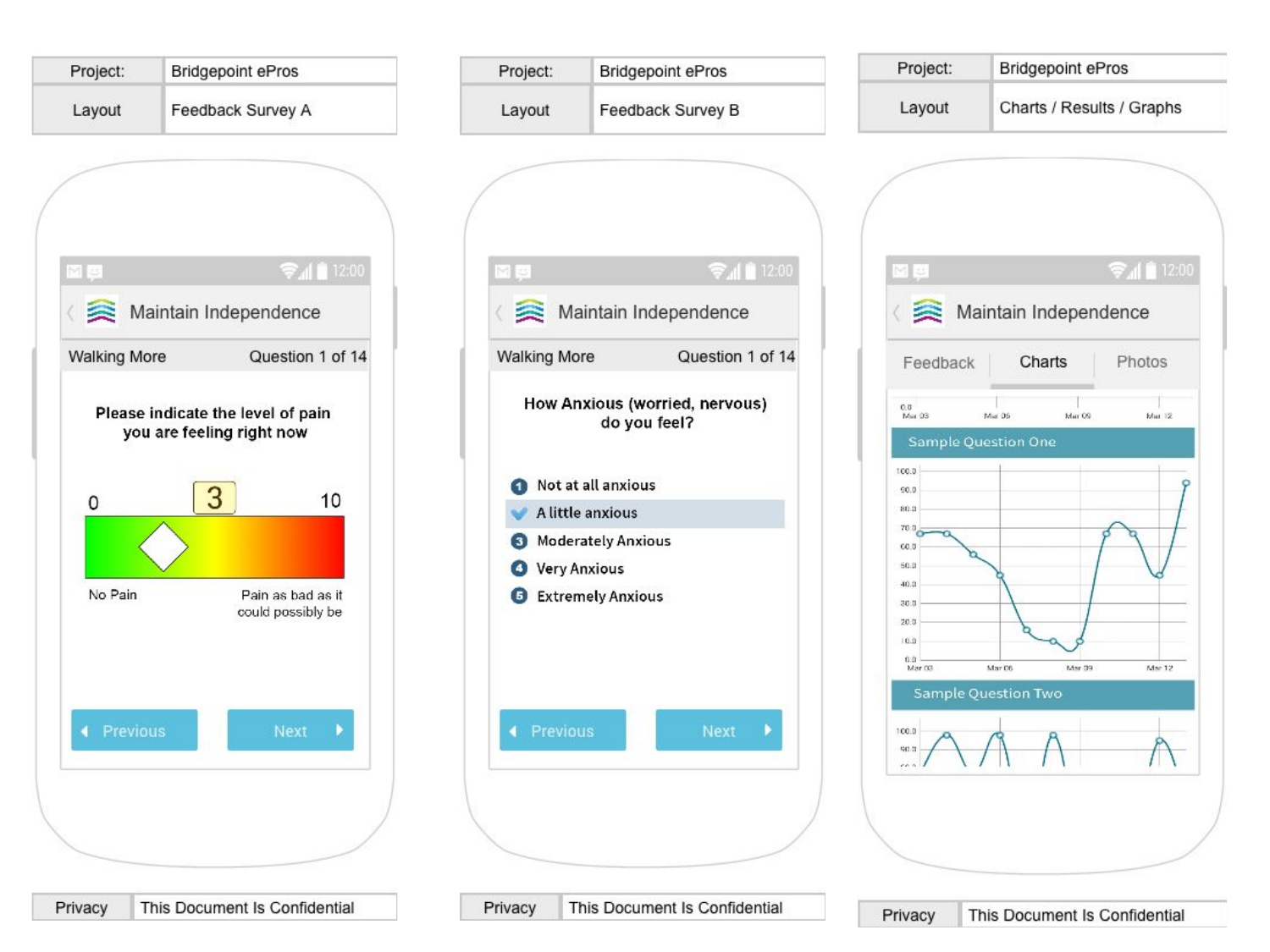

Supplement: Multimedia Appendix 1 [file resprot_v5i2e126_app1.pptx]
