# Supplementary material for: Supporting Goal-Oriented Primary Health Care for Seniors with Complex Care Needs Using Mobile Technology: Evaluation and Implementation of the Health System Performance Research Network, Bridgepoint Electronic Patient Reported Outcome Tool
Source: JMIR Res Protoc. 2016 Jun 24;5(2):e126. doi: 10.2196/resprot.5756 (PMC4938886; doi:10.2196/resprot.5756)
Supplement: Multimedia Appendix 2 [file resprot_v5i2e126_app2.pdf]

Review Type/Type d'évaluation: Committee Member 1/Membre de comité 1
Name of Applicant/Nom du chercheur: STEELE GRAY, Carolyn Alice
Application No./Numéro de demande: 348362
Agency/Agence: CIHR/IRSC
Competition/Concours: 2015-06-03 Operating Grant: eHealth Innovations Initiative: eHealth Innovation Partnership Program (eHIPP)/Subvention de fonctionnement: Initiative Innovations en cybersanté : Programme de partenariats pour l’innovation en cybersanté (PPIC)
Committee/Comité: eHealth Innovation Partnership Program (eHIPP): Support of Seniors/Programme de partenairiats pour l'innovation en cybersanté-L'appui awx personnes âgéess
Title/Titre: Supporting Goal-Oriented Primary Health Care for Seniors with Complex Care Needs using Mobile Technology: Evaluation and implementation of the HSPRN-Bridgepoint ePRO Tool.

Assessment/Évaluation:

Comments

Proposal explicit in addressing evaluation criteria.

Planned Intervention: A well-developed goal-setting tool, beyond "state of art" intervention to agree and monitor progress towards agreed goals with older people with complex needs and their care-givers. Based upon useful user-centred iterative design processes: appendices provide screen-shots of prototype.. Generic outcomes well developed: Quality-of-life, experience of care, attainment of goals, cost-effectiveness: convincing as broad outcomes. Some potential to integrate into primary care services.

eHealth Innovation Evaluation: appropriate approach to confirm design of intervention in real-world settings, yet **highly diverse target population** of older people with two or more chronic conditions. Phased study with 1,2,3 done and 4 under-way, Application for next phase: suggests low risk proposal. Bias acknowledged and well-addressed. Includes a convincing cost-effectiveness component. Documents prepared for data collection, participant information & consent (ethics addressed)

Team: good team with high likelihood of success, includes international partners, led by industry partner,

Budget: no comment. CAN$91,387 in year 1.

Review Type/Type d'évaluation: Committee Member 2/Membre de comité 2
Name of Applicant/Nom du chercheur: STEELE GRAY, Carolyn Alice
Application No./Numéro de demande: 348362
Agency/Agence: CIHR/IRSC
Competition/Concours: 2015-06-03 Operating Grant: eHealth Innovations Initiative: eHealth Innovation Partnership Program (eHIPP)/Subvention de fonctionnement: Initiative Innovations en cybersanté : Programme de partenariats pour l'innovation en cybersanté (PPIC)
Committee/Comité: eHealth Innovation Partnership Program (eHIPP): Support of Seniors/Programme de partenairiats pour l'innovation en cybersanté-L'appui awx personnes âgéess
Title/Titre: Supporting Goal-Oriented Primary Health Care for Seniors with Complex Care Needs using Mobile Technology: Evaluation and implementation of the HSPRN-Bridgepoint ePRO Tool.

Assessment/Évaluation:

Determining how to engage older adults in taking greater responsibility for their health care is a very important goal in today's world where most older adults have been indoctrinated in believing that the health care system knows best.

This approach has great merit for the younger older adults of today and may be of benefit to some of the mid and old older adults.

A paradigm shift toward shared decision making and responsibility taking is essential to future health care therefore this may be a good way to make that transition to goal oriented primary health care.

Good combination of researchers, practitioners and technology.

Review Type/Type d'évaluation: Committee Member 3/Membre de comité 3
Name of Applicant/Nom du chercheur: STEELE GRAY, Carolyn Alice
Application No./Numéro de demande: 348362
Agency/Agence: CIHR/IRSC
Competition/Concours: 2015-06-03 Operating Grant: eHealth Innovations Initiative: eHealth Innovation Partnership Program (eHIPP)/Subvention de fonctionnement: Initiative Innovations en cybersanté : Programme de partenariats pour l’innovation en cybersanté (PPIC)
Committee/Comité: eHealth Innovation Partnership Program (eHIPP): Support of Seniors/Programme de partenairiats pour l'innovation en cybersanté-L'appui awx personnes âgéess
Title/Titre: Supporting Goal-Oriented Primary Health Care for Seniors with Complex Care Needs using Mobile Technology: Evaluation and implementation of the HSPRN-Bridgepoint ePRO Tool.

Assessment/Évaluation:

Planned Intervention:
Building on successful development of an eHealth tool to allow patients with complex needs to create and monitor their own goals for care, this project aims to use the tool to enable monitoring, self management, and shared decision-making. Seniors seldom present with only one health problem and this innovation takes that into account. It will address quality of life issues for seniors with complex care needs. The technology has been developed and is already at TRL of 7. It is build using a tested platform . There is a high probability that the ePRO can improve self-management, patient experience and quality of life. Patients have been involved from the beginnings of development of this tool and have found it effective to assist with goal setting and monitoring. There is a willingness and interest on the part of patient participants to continue their involvement with co-development of this project. Change management has been addressed with a very comprehensive plan that is supported by team members with strong change management skills. It follows Canada Health Infoway’s Change Management Framework to support adoption of the ePRO tool. The involvement of international partners indicates a perception of usability and value and will greatly aid with scalability and adoption.

eHealth Innovation Evaluation:
The research questions are clear and reflect the desired outcomes: improved self-management, patient experience and quality of life, and cost effectiveness. These outcomes are very relevant and important to seniors such as myself. The sample size has been calculated to reflect 80% power derived from a power calculation, with 660 participants in all. The novel approach of utilizing embedded case studies to collect unique differences will strengthen the evaluation through identification of outcomes which may not have been anticipated. Approaches to bias control, cost-effectiveness evaluation and analysis including sex/gender analysis appear well thought out and complete.

Team:
Many of the team have been involved in the development process that preceded this research and as such have already worked together towards a successful product. Patient involvement in the team is evident and essential in this patient-centered application. Skills in innovation, eTechnology, and seniors care are all apparent in the team as is the capacity to complete successful quality evaluation.

**Review Type/Type d'évaluation:**	Committee Member 4/Membre de comité 4
**Name of Applicant/Nom du chercheur:**	STEELE GRAY, Carolyn Alice
**Application No./Numéro de demande:**	348362
**Agency/Agence:**	CIHR/IRSC
**Competition/Concours:**	2015-06-03 Operating Grant: eHealth Innovations Initiative: eHealth Innovation Partnership Program (eHIPP)/Subvention de fonctionnement: Initiative Innovations en cybersanté : Programme de partenariats pour l'innovation en cybersanté (PPIC)
**Committee/Comité:**	eHealth Innovation Partnership Program (eHIPP): Support of Seniors/Programme de partenairiats pour l'innovation en cybersanté-L'appui awx personnes âgéess
**Title/Titre:**	Supporting Goal-Oriented Primary Health Care for Seniors with Complex Care Needs using Mobile Technology: Evaluation and implementation of the HSPRN-Bridgepoint ePRO Tool.

Assessment/Évaluation:

Proposal Number - #348362

Nothing further to add to CSO notes

Planned Intervention:

eHealth Innovation:

Team:

Budget:
